# Supplementary material for: Zfy genes are required for efficient meiotic sex chromosome inactivation (MSCI) in spermatocytes
Source: Hum Mol Genet. 2016 Oct 13;25(24):5300–10. doi: 10.1093/hmg/ddw344 (PMC5418838; doi:10.1093/hmg/ddw344)
Supplement: Supplementary Data [file ddw344_Supp.zip › ddw344-suppl_data/Figure_S1.docx]

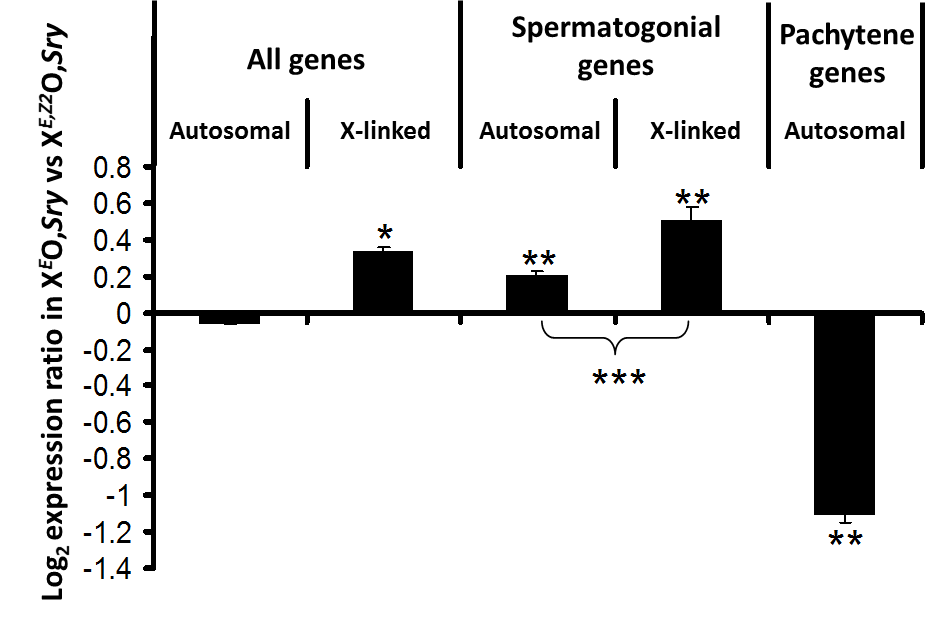


**Figure S1**

Bar chart showing the average log_2_ expression ratio in X*^E^*O*Sry* versus X*^E,Z2^*O*Sry* for all genes, spermatogonia-specific genes and pachytene-specific genes. For this analysis, ‘spermatogonia-specific’ and ‘pachytene-specific’ were defined as those showing at least a tenfold decrease in transcript abundance between B spermatogonia and pachytene spermatocytes in Namekawa et al (2006). This was to ensure that autosomal genes chosen as a comparator set are an appropriate match for the X chromosome genes, which are transcribed in B spermatogonia but silenced by MSCI during pachynema.

* The X chromosome is globally upregulated in X*^E^*O*Sry* (p = 4.5 x 10^-39^)

** Autosomal and X spermatogonia-specific genes are upregulated and autosomal pachytene-specific genes are downregulated in X*^E^*O*Sry* (p < 1 x 10^-7^ for each subset).

*** X spermatogonia-specific genes are more strongly upregulated than autosomal spermatogonia-specific genes in X*^E^*O*Sry* (p = 4.80 x 10^-8^).
