# Supplementary material for: Zfy genes are required for efficient meiotic sex chromosome inactivation (MSCI) in spermatocytes
Source: Hum Mol Genet. 2016 Oct 13;25(24):5300–10. doi: 10.1093/hmg/ddw344 (PMC5418838; doi:10.1093/hmg/ddw344)
Supplement: Supplementary Data [file ddw344_Supp.zip › ddw344-suppl_data/Figure_S2.docx]

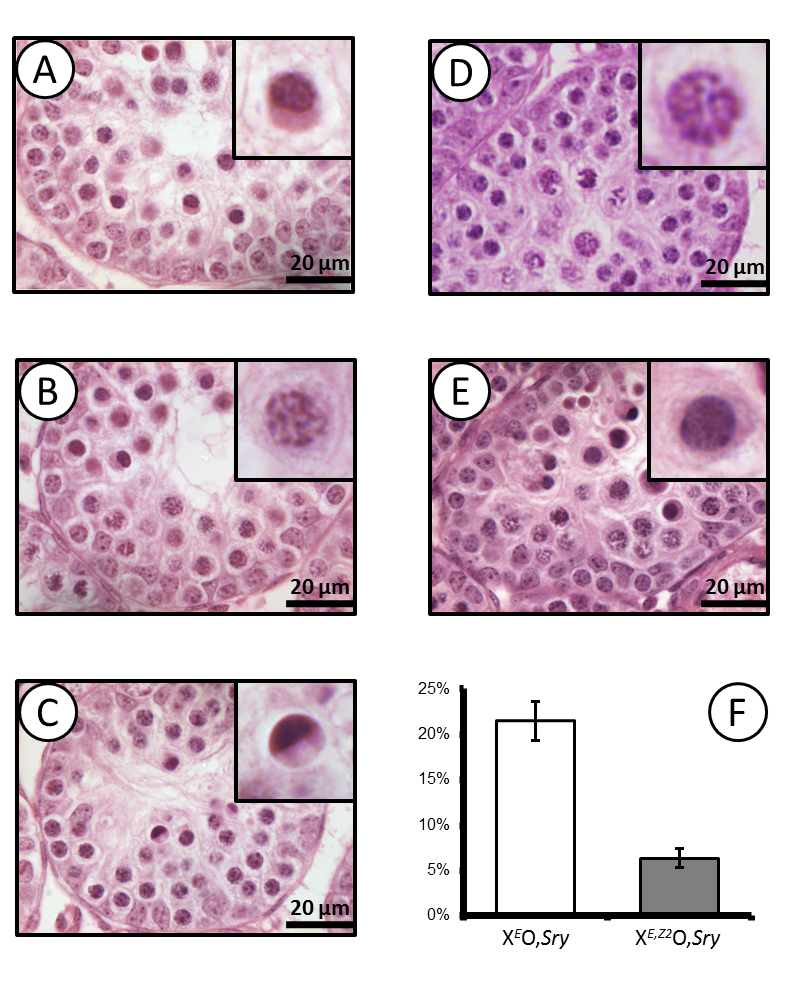


**Figure S2**
Apoptosis was studied by H&E staining of testis sections at 15dpp. Scale bar = 20μm, insets = 3x magnification. In X*^E^*O,*Sry* testes, pachytene cell apoptosis was observed from stage IV onwards, however this did not represent a typical stage IV block as a substantial proportion of cells survived past this stage. Pachytene cells that survived stage IV continued to die over subsequent stages, however survivors were observed as late as stage IX, the latest stage present at 15dpp. In some tubules, particularly at stages IV and V, we observed abnormal spermatocytes that had homogeneous chromatin and where the cells appeared to be no longer in prophase, but which were not yet apoptotic. The total proportion of apoptotic spermatocytes across all stages was significantly higher in X*^E^*O,*Sry* testes than in X*^E,Z2^*O,*Sry* testes at this age.

**A:** stage IV tubule with almost all cells apoptotic: inset = dying pachytene cell.

**B:** stage IV tubule with apoptotic pachytenes in upper left and surviving pachytenes at the bottom: inset = surviving pachytene cell.

**C:** stage VI tubule with mostly apoptotic pachytene cells: inset = dying pachytene cell.

**D:** stage IX tubule with multiple surviving pachytene cells: inset = surviving pachytene cell.

**E:** stage V tubule with a mix of surviving, abnormal and apoptotic pachytene spermatocytes: inset = abnormal cell showing homogeneous chromatin without other hallmarks of apoptosis.

**F:** histogram showing the proportion of dying pachytene cells in both genotypes at 15dpp, p=0.0029 by Student t test after arcsin transformation of percentage data.
