# Supplementary material for: Zfy genes are required for efficient meiotic sex chromosome inactivation (MSCI) in spermatocytes
Source: Hum Mol Genet. 2016 Oct 13;25(24):5300–10. doi: 10.1093/hmg/ddw344 (PMC5418838; doi:10.1093/hmg/ddw344)
Supplement: Supplementary Data [file ddw344_Supp.zip › ddw344-suppl_data/Inventory_of_Supplements.docx]

**Inventory of Supplements

Figure S1:** Bar chart showing the mean log_2_ expression ratio in X*^E^*O*Sry* versus X*^E,Z2^*O*Sry* for all genes, spermatogonia-specific genes and pachytene-specific genes.

**Figure S2:** Supplementary histology images for X*^E^*O*Sry* testes at 15 dpp showing cells surviving past the stage IV checkpoint and other abnormal cells.

**Table S1:** Normalised array data and processing pipelines.

**Table S2:** Cell count data from the RNA FISH experiments.

**Table S3:** Tubule counts and cell counts for analysis of apoptosis.

**Table S4:**  Cell counts for analysis of early meiotic progression and synaptonemal complex assembly

**Table S5:** Tubule counts for analysis of meiotic progression by BrdU staining.

**Supplementary Methods:** Inventory of BAC probes used for RNA FISH experiments, antibodies used for immunostaining, and primers used for qPCR.
