# Supplementary material for: Zfy genes are required for efficient meiotic sex chromosome inactivation (MSCI) in spermatocytes
Source: Hum Mol Genet. 2016 Oct 13;25(24):5300–10. doi: 10.1093/hmg/ddw344 (PMC5418838; doi:10.1093/hmg/ddw344)
Supplement: Supplementary Data [file ddw344_Supp.zip › ddw344-suppl_data/Supplementary_Methods.docx]

**SUPPLEMENTAL EXPERIMENTAL PROCEDURES (REAGENT LIST)**

**BAC clones used to prepare RNA FISH probes:**

*Adam3* : RP24‑103I8 (CHORI)

*GM773* : RP23‑121P2 (CHORI)

*Magea2/5* : RP23‑169B15 (CHORI)

*Scml2* : RP24-204O18 (CHORI)

*Zfx* : BMQ‑372M23 (Research Genetics).

**Primary antibodies used for cell staging in RNA FISH experiments on spread cells**

Rabbit anti-SYCP3 (1:100, Abcam: ab15093)

Mouse monoclonal anti-γH2AFX (1:100, Upstate: 16–193)

Guinea pig anti-HORMAD2 (1:200, Cloutier et al., 2015a).

**Primary and secondary antibodies used for immunofluorescence experiments on testis sections**

Rabbit anti-MVH, (1:200, Toyooka et al., 2000)

Mouse monoclonal anti-γH2AFX, (1:500, Upstate: 05–636).

Chicken anti-rabbit Alexa 488 (1:500, Molecular Probes)

Chicken anti-mouse Alexa 594 (1:500, Molecular Probes)

**Primers used for quantitative RT‑PCR**

| Gene | Primer ID | Primer sequence | Amplicon  size |
| --- | --- | --- | --- |
| *Rhox3a* | *Rhox3a* -F | ggagagagtgaccaggctga | 165bp |
|  | *Rhox3a* -R | ttcattcacacccatccatc |  |
| *Atp7a* | *Atp7a* -F | tgaccttctggatgttgtgg | 162bp |
|  | *Atp7a* -R | gatcccatccagggttgtaa |  |
| *Zfx* | *Zfx* -F | gcacttgccctgaagtcatc | 273bp |
|  | *Zfx*-R | cagctgcagcatcctcct |  |
| *Tdrd6* | *Tdrd6*-F | tccaaacagtgggaaaggac | 176bp |
|  | *Tdrd6*-R | gtagcaccgggcttgttaaa |  |
| *Piwil2* | *Piwil2*-F | aatggtactcgagggtggtg | 153bp |
|  | *Piwil2*-R | ggccatcagacactccatct |  |
| *Mad2l2* | *Mad2l2*-F | attctctatgtgcgcgaggt | 133bp |
|  | *Mad2l2*-R | gaggtttgacgcagtggagt |  |
| *β*-*actin* | *β*-*actin*-F | GGCACCACACCTTCTACAATG | 352bp |
|  | *β*-*actin*-R | GTGGTGGTGAAGCTGTAGCC |  |

**Supplemental References**

Toyooka, Y., Tsunekawa, N., Takahashi, Y., Matsui, Y., Satoh, M., and Noce, T. (2000) Expression and intracellular localization of mouse Vasa-homologue protein during germ cell development. Mech Dev *93*, 139-149.
