# Supplementary material for: Zfy genes are required for efficient meiotic sex chromosome inactivation (MSCI) in spermatocytes
Source: Hum Mol Genet. 2016 Oct 13;25(24):5300–10. doi: 10.1093/hmg/ddw344 (PMC5418838; doi:10.1093/hmg/ddw344)
Supplement: Supplementary Data [file ddw344_Supp.zip › ddw344-suppl_data/2016-09-13_Zfy2_HMG-resubmission.doc]

***Zfy* genes are required for efficient meiotic sex chromosome inactivation (MSCI) in spermatocytes**

**Running title: A role for *Zfy* in X-Y silencing**

Nadège Vernet,^1,2^ Shantha K. Mahadevaiah,^1,3^ Dirk G. de Rooij^4^, Paul S. Burgoyne^1,3^ and Peter J. I. Ellis.^5,6,*^

^1^	Division of Stem Cell Biology and Developmental Genetics, MRC National Institute for Medical Research, Mill Hill, London NW7 1AA, UK.

^2^	Department of Functional Genomics and Cancer, Institut de Génétique et de Biologie Moléculaire et Cellulaire, 67404 Illkirch Cedex, France.

^3^	Division of Stem Cell Biology and Developmental Genetics, The Francis Crick Institute, Mill Hill Laboratory, The Ridgeway, London NW7 1AA, UK.

^4^	Reproductive Biology Group, Division of Developmental Biology, Department of Biology, Faculty of Science, Utrecht University, Utrecht, The Netherlands

^5^	Department of Pathology, University of Cambridge, Cambridge CB2 1QP, UK.

^6^	School of Biosciences, University of Kent, Canterbury CT2 7NZ, UK.

^*^	Corresponding author: [p.j.i.ellis@kent.ac.uk](mailto:p.j.i.ellis@kent.ac.uk)

**Key words: Meiosis, meiotic silencing, Zfy, sex chromosomes, spermatogenesis, apoptosis**

**Abstract**

During spermatogenesis, germ cells that fail to synapse their chromosomes or fail to undergo meiotic sex chromosome inactivation (MSCI) are eliminated via apoptosis during mid-pachytene. Previous work showed that Y-linked genes *Zfy1* and *Zfy2* act as “executioners” for this checkpoint, and that wrongful expression of either gene during pachytene triggers germ cell death. Here, we show that in mice, *Zfy* genes are also necessary for efficient MSCI and the sex chromosomes are not correctly silenced in *Zfy*-deficient spermatocytes. This unexpectedly reveals a triple role for *Zfy* at the mid-pachytene checkpoint in which *Zfy* genes first promote MSCI, then monitor its progress (since if MSCI is achieved, *Zfy* genes will be silenced), and finally execute cells with MSCI failure. This potentially constitutes a negative feedback loop governing this critical checkpoint mechanism.

**Introduction**

In mammals, quality control during gametogenesis is far more stringent in males than in females, and consequently the vast majority of aneuploidies arise via non-disjunction during oogenesis, rather than spermatogenesis . The proximate mechanism for this difference is the efficient apoptotic elimination of developing gametes carrying univalent (unpaired) chromosomes that occurs during male, but not female meiosis . This elimination involves two separate checkpoints: a checkpoint operating during mid-pachytene that monitors both the synapsis of homologous chromosomes and meiotic sex chromosome inactivation (MSCI) , and a spindle assembly checkpoint (SAC) operating at metaphase of the first meiotic division . *Zfy* genes have been implicated in the control of apoptosis at both of these checkpoints (Royo et al., 2010; Vernet et al., 2011).

*Zfy* genes are Y-linked transcription factors conserved throughout eutherian mammals . In humans, a single *ZFY* gene expresses two major splice variants: a full length version which shows transactivation ability in a yeast reporter system , and a short version which lacks a key acidic domain and has no detectable transactivation activity. In the mouse, there are two paralogous copies, *Zfy1* and *Zfy2*: *Zfy1* preferentially expresses the short (inactive) isoform, while *Zfy2* predominantly expresses the long (active) isoform . Moreover, the transactivation activity of full-length *Zfy2* is substantially higher than full-length *Zfy1* , suggesting that the bulk of *Zfy* transactivation activity in the mouse is supplied by *Zfy2*.

Like all protein-coding genes in the non-pairing regions of the sex chromosomes, *Zfy* genes are subject to meiotic sex chromosome inactivation (MSCI) in pachytene cells . During MSCI, the unsynapsed axes of the sex chromosomes become condensed and transcriptionally inactive, forming the “sex body” marked by the phosphorylated form of histone H2AX (γH2AX) . When present as autosomal transgenes, *Zfx/y* genes evade MSCI and are expressed ectopically during pachytene. In the cases of both *Zfy1* and *Zfy2* this triggers germ cell apoptosis during stage IV of the spermatogenic cycle (Royo et al., 2010). Extensive germ cell apoptosis at the same tubule stage is seen in other models where MSCI, and in particular Y chromosome silencing is impaired (e.g. XYY males, *H2afx* *-/-, Hormad2 -/-*) , indicating that successful silencing of *Zfy1* and *Zfy2* is an absolute requirement for pachytene progression. This has therefore been termed an “MSCI checkpoint” mediated in part by overexpression of *Zfy* genes during pachytene . In many model systems with autosomal synapsis failure (e.g*. Dmc* -/-, *Hormad1 -/-*, *Msh5* -/-, *Spo11* -/-) there is substantial apoptosis during tubule stage IV which was initially interpreted as a “synapsis checkpoint” paralleling the mid-pachytene synapsis checkpoint known to operate in yeast . However, it is now known that in these mouse models, the failure of synapsis leads secondarily to failure of MSCI , and hence the MSCI checkpoint and the synapsis checkpoint are now widely believed to reflect different aspects of the same process.

Turning to the spindle assembly checkpoint (SAC), a convenient model system to investigate the underlying mechanisms is the response to the univalent X chromosome in sex-reversed XO mice transgenic for *Sry* (conferring maleness) and *Eif2s3y* (necessary for spermatogonial proliferation) – we refer to these hereafter as X*^E^*O,*Sry* males . In X*^E^*O,*Sry* spermatocytes, the univalent X chromosome triggers cell cycle arrest at the first meiotic metaphase, however the fate of the arrested cells is dependent on the presence of *Zfy2*. In the absence of *Zfy2*, germ cells recover and complete the first meiotic division despite X univalence, and arrest again as interphasic secondary spermatocytes. These secondary spermatocytes subsequently differentiate into diploid spermatids before undergoing apoptosis. In contrast, when the *Zfy2* deficiency is complemented by an X-linked *Zfy2* transgene (we refer to these as X*^E,Z2^*O,*Sry* males), the germ cells undergo prompt apoptosis following the initial arrest at the first meiotic metaphase (MI) . There is therefore a requirement for *Zfy2* in enabling apoptosis at the MI SAC.

The mechanism by which *Zfy2* can regulate apoptosis at the MI SAC is puzzling, since as covered above it is not expressed – indeed its expression is not tolerated! – in pachytene cells. The original objective of the present study, therefore, was to determine the transcriptional consequences of *Zfy2* deficiency during pachytene by comparison of X*^E^*O,*Sry* and X*^E,Z2^*O,*Sry* males at 17.5 days *post partum* (dpp), during the first wave of pre-pubertal germ cell development. Our working hypothesis was that *Zfy2* expression prior to pachytene activates pro-apoptotic genes, and that these downstream effects persist until MI and thus enable apoptosis at the MI SAC checkpoint. Surprisingly, our analysis showed that in X*^E^*O,*Sry* males at 17.5 dpp there is a widespread failure of MSCI and overexpression of X-linked genes. We therefore determined whether there is continued MSCI leakage at later ages in this model, which also proved to be the case. Finally, we carried out a preliminary analysis of the downstream responses to MSCI leakage in X*^E^*O,*Sry* males both during the first wave and at later ages, in order to understand the dual roles of *Zfy* in promoting efficient MSCI and in promoting the apoptotic response to MSCI failure. Based on our findings, we propose that *Zfy* genes may function as a negative feedback “sensor” system that regulates the onset of MSCI, monitors its progress and finally promotes the execution of cells with MSCI failure.

**Results**

**Lack of *Zfy* leads to widespread X upregulation and downregulation of pachytene-specific genes at 17.5 dpp**

We carried out microarray expression analysis of whole testis tissue from X*^E^*O,*Sry* (lacking *Zfy* genes), and X*^E,Z2^*O,*Sry* (with *Zfy2* restored) at 17.5 dpp. At this age, the most advanced spermatocytes in the testis are in late pachytene or early diplotene, just prior to meiosis I and activation of the SAC checkpoint. Thus, it is before both the MI arrest and apoptosis seen in X*^E,Z2^*O,*Sry* and the accumulation of arrested secondary spermatocytes in X*^E^*O,*Sry*.

The microarray results unexpectedly showed a preferential up-regulation of X-linked transcripts and down-regulation of autosomal transcripts in response to *Zfy* deficiency (**Figure 1A**). This held true regardless of whether we focused on statistically significant transcripts, or on all transcripts changing by more than 1.5 fold regardless of significance (**Supplementary** **Table S1**).

To test whether the observed difference in X expression was related to the absence of *Zfy* genes in X*^E^*O,*Sry*, or an off-target effect of the *Zfy2* transgene in X*^E,Z2^*O,*Sry*, we compared the expression data to previously-obtained data from 17.5 dpp normal XY males (**Figure 1B**) . Although there was marked variation in average X expression between individual replicates at this age, there was a highly significant upregulation of the X chromosome in X*^E^*O,*Sry* males relative to wild type XY males, and this was largely corrected in X*^E,Z2^*O,*Sry*. There was a slight but significant residual X upregulation seen in X*^E,Z2^*O,*Sry* relative to wild type XY males (p = 0.0013), which may be due to the additional presence of *Zfy1* in wild type males.

To determine the normal cellular distribution of the transcripts altered by *Zfy* deficiency, we compared our results to published data from separated germ cells [GSE4193] . A scatter plot of X*^E^*O,*Sry* : X*^E,Z2^*O,*Sry* log_2_ ratio (our new data) versus the pachytene : B spermatogonia log_2_ ratio (calculated from the Namekawa et al. dataset) shows that there is a good correlation between these measures (**Figure 1C**, r^2^ = 0.5373). That is, transcripts which in wild type males are more strongly expressed in pachytene spermatocytes than in B spermatogonia (high P:B ratio) are downregulated in response to *Zfy* deficiency, and *vice versa*. We conclude that in *Zfy*-deficient testes, there is a global upregulation of the X chromosome that is associated with a deficiency of pachytene-specific spermatocyte transcripts as a proportion of total testis RNA, and a corresponding excess of spermatogonia-specific transcripts.

**MSCI “leakage” in X*^E^*O,*Sry* spermatocytes at 17.5 dpp is associated with apoptosis during pachytene**

Since X-linked genes are in general transcriptionally active in spermatogonia and inactive in spermatocytes, we considered whether the upregulation of X transcripts in X*^E^*O*Sry* could be entirely explained by the shift in the balance between spermatogonial and spermatocyte transcriptomes. Importantly, X-linked spermatogonia-specific transcripts were more strongly upregulated than autosomal spermatogonia-specific transcripts (**Supplementary Figure S1**). This indicates that the increase in X-linked transcript abundance cannot be explained solely by an increase in the proportion of spermatogonia in the testis, since this would affect all spermatogonia-specific genes irrespective of chromosomal location.

The combination of upregulation of X-linked genes and a shift in the proportion of spermatogonia-specific vs pachytene-specific transcripts was therefore strongly suggestive of MSCI failure , since MSCI failure is expected to lead to germ cell loss during pachytene. We therefore predicted that the upregulation of X genes and downregulation of autosomal pachytene-specific genes should initiate around 13 dpp when pachytene cells first appear in the testis, and the autosomal downregulation should increase in severity over subsequent days as the proportion of pachytene cells increases in normal testes, and this was confirmed by qPCR (**Figure 1D**).

As a second measure of MSCI leakage during the first wave of spermatogenesis, we performed RNA FISH experiments to determine whether the X-linked gene *Scml2* was correctly silenced. *Scml2* is an X-linked gene near the pseudoautosomal region (PAR) boundary, is one of the last genes to become fully silenced during normal meiosis, and is therefore a sensitive indicator of MSCI failure. Initial experiments showed that a γH2AX-containing sex body was formed in spermatocytes of all genotypes including X*^E^*O,*Sry*, and so in these analyses we identified spermatocytes by immunostaining for γH2AX together with DAPI staining of DNA. It is possible that some spermatocytes failed to form an identifiable γH2AX-positive sex body and were excluded from our analysis. Since failure to form a γH2AX-positive sex body is universally associated with MSCI failure, this means that our results will therefore be a minimum estimate of the proportion of spermatocytes with MSCI leakage in each genotype. Leaky transcription of *Scml2* was observed in 12-42% of X*^E^*O,*Sry* pachytene spermatocytes at 17-18 dpp, compared to 5-6% in X*^E,Z2^*O,*Sry* spermatocytes and 2-3% in XY wild type spermatocytes.

MSCI leakage is expected to lead to spermatocyte apoptosis at the mid-pachytene stage IV checkpoint. We therefore used hematoxylin and eosin (H&E) staining of testis sections to quantitate the level of apoptosis in X*^E^*O*Sry* testes at 15 dpp (**Figure 2** upper panels and **Table 1**), this being the earliest time point at which autosomal pachytene-specific gene downregulation was observed by qPCR. Consistent with the gene expression data, there was a borderline significant increase in the number of tubules containing apoptotic cells in X*^E^*O,*Sry*, together with a highly significant increase in the number of apoptotic cells per tubule. Although most of the tubules with high levels of apoptosis in X*^E^*O,*Sry* were judged to be in stage IV, this did not constitute a complete stage IV block and both surviving and apoptotic pachytene spermatocytes were observed throughout subsequent stages. We also noticed a population of cells in which the chromatin was homogeneous (suggestive of prophase exit) but which were not yet judged to be apoptotic (**Supplementary Figure S2**). Owing to the difficulty of generating these genotypes, we were unable to generate enough sample material to quantitate the percentage of apoptosis in each individual tubule stage.

**MSCI “leakage” in X*^E^*O,*Sry* spermatocytes is also present at later ages**

The finding of leaky MSCI and pachytene cell death in X*^E^*O,*Sry* testes during the first wave of spermatogenesis presents a paradox in that our previous work has documented a marked accumulation of diploid spermatids in X*^E^*O,*Sry* males at 30-31 dpp (Vernet et al., 2011). This accumulation indicates that many cells survive up to and beyond the meiotic cell divisions. We considered it possible therefore that the MSCI leakage might be restricted to the first wave of spermatogenesis. To test this, we extended our investigation of the MSCI leakage to the 30-31 dpp timepoint used in our studies of the SAC. Direct expression analysis of these genotypes is not feasible at this age, because expression profiles from whole testis are confounded by the accumulation of arrested cells in X*^E^*O,*Sry* that are removed at the SAC checkpoint by apoptosis in X*^E,Z2^*O,*Sry*. Therefore, in these older animals we assayed MSCI leakage using RNA FISH only (**Figure 3** and **Supplementary** **Table S2).** As with the 17.5 dpp males, spermatocytes were identified by γH2AX and DAPI staining. In some experiments, spermatocytes were further divided into early and late stages by RNA FISH for *Adam3*, an autosomal gene expressed exclusively in late pachytene / diplotene spermatocytes.

RNA FISH for *Scml2* at 30-31 dpp confirmed that there is MSCI leakage in X*^E^*O,*Sry* at this age relative to WT testes (n=3 per genotype). Transgenic replacement of either *Zfy1* or *Zfy2* greatly reduces the MSCI leakage, (n=3 per genotype). X^Sxra^O males (n=3), which have both *Zfy1* and *Zfy2* , also showed very little MSCI leakage. Aggregating all our experiments together, the proportion of late pachytene spermatocytes (i.e. cells positive for *Adam3*) with MSCI leakage (i.e. where the cells were also positive for *Scml2*) was 66/766 cells in X*^E^*O,*Sry* males*,* 15/665 cells in males transgenic for *Zfy2*, 3/403 cells in males transgenic for *Zfy1* and 2/403 cells in X^Sxra^O males (**Supplementary Table S2**). All three “rescued” genotypes are significantly different from X*^E^*O,*Sry* (p << 0.01 by chi-squared test in all cases), however the “rescued” genotypes are not significantly different from each other after multiple testing correction. We therefore conclude that both *Zfy* genes are able promote MSCI at 30-31 dpp, but our data do not show which gene copy is more effective.

Finally, we examined young adult testes (6 weeks to 2 months *post partum*, **Figure 4**) by RNA FISH. Again, we observed MSCI leakage in early pachytene *Adam3*-negative spermatocytes in X*^E^*O,*Sry*, which was reduced but not absent in late pachytene / diplotene *Adam3*-positive spermatocytes. Transgenic replacement of *Zfy2* abolished the MSCI leakage in both early and late spermatocytes at this age. In this experiment we also investigated three further X-linked genes; *Zfx*, *GM773* and *Mage2a+5*. There was a strong per-cell correlation between X-linked genes in terms of MSCI escape: e.g. all cells positive for *Magea 2/5* leakage were also positive for *Scml2* leakage. This indicates that in young adult X*^E^*O,*Sry* spermatocytes the leaking X genes are not independent of each other. Together with the fact that *Scml2* - which showed the highest levels of MSCI leakage - is known to be slow to achieve MSCI in normal cells, this suggests that the MSCI leakage in young adult X*^E^*O,*Sry* testes may represent a delayed imposition of MSCI, rather than stochastic breakthrough activity of individual X genes.

**The MSCI leakage in X*^E^*O,*Sry* at 30-31 dpp does not lead to apoptosis of pachytene spermatocytes**

The finding of ongoing MSCI leakage in older X*^E^*O,*Sry* testes was surprising (as noted above) since we know that at this age germ cells are able to survive up to and beyond the first meiotic division. Moreover, in our RNA FISH data, at both 30-31 dpp and at two months old, for every X gene examined we were able to find cells positive for both *Adam3* and the X gene of interest, indicating that at least some X*^E^*O,*Sry* spermatocytes are able to survive past the stage IV checkpoint despite ongoing MSCI leakage. We therefore used three independent methods to quantitate apoptosis during pachytene in males at 30-31 dpp (see Methods). We found no significant difference between genotypes either in the proportion of tubules with apoptotic pachytene cells or in the number of apoptotic cells per tubule, by H&E staining or by TUNEL staining. In stage XII tubules at this age, while the number of apoptotic MI metaphase spermatocytes per tubule was significantly increased in X*^E,Z2^*O,*Sry* relative to X*^E^*O,*Sry* due to the reinstatement of the SAC by the *Zfy2* transgene, there was no difference between the genotypes in the total number of cells entering metaphase I (**Table 1**).

We conclude that in X*^E^*O,*Sry* testes, the MSCI leakage observed by gene expression analysis of juvenile males is associated with increased pachytene apoptosis during the first wave of spermatogenesis, while the MSCI leakage observed by RNA FISH analysis at 30-31dpp does not lead to a detectable increase in apoptosis.

**X*^E^*O,*Sry* males show no alteration either in early meiotic progression or in the overall length of meiotic prophase**

Since the cells with MSCI leakage do not appear to undergo apoptosis in older X*^E^*O,*Sry* males, we sought to determine whether *Zfy* deficiency and MSCI leakage had any other effects on either early meiotic progression or on the overall length of meiotic prophase.

The first of these was assayed by immunostaining for SCP3, HORMAD2 and γH2AX in order to define different stages of synaptonemal complex assembly and sex body formation (**Supplementary Table S4**). This showed no change between X*^E^*O,*Sry* and X*^E,Z2^*O,*Sry*.

The second was assayed by BrdU staining of developing testes (**Figure 5** and **Supplementary Table S5**). Following a single injection of BrdU at 18-21 dpp, BrdU incorporated during early spermatogonial divisions is diluted in subsequent rounds of DNA replication and is not detectable. This protocol thus traces the fate of those germ cells undergoing the final two rounds of spermatogonial DNA replication and division at the time of injection. In normal XY testes, at 12 days after the BrdU injection, these germ cells have completed one full round of the spermatogenic cycle, and are detected as pachytene spermatocytes of stage IX to XII. The stage distribution of BrdU-positive tubules present in wild type XY males, X*^E^*O,*Sry* males and X*^E,Z2^*O,*Sry* males at 12 days post injection revealed a unimodal distribution in all cases, with no delay in meiotic progression either between X*^E^*O,*Sry* and X*^E,Z2^*O,*Sry* or relative to XY wild type.

**Discussion**

***Zfy* genes are required for efficient meiotic sex chromosome inactivation**

In this study, we investigated the transcriptional and phenotypic consequences of *Zfy* gene deficiency during pachytene in the context of X*^E^*O,*Sry* sex-reversed male mice. These are males that completely lack Y chromosomal gene content other than transgenes for *Sry* (promoting maleness), *Eif2s3y* (promoting spermatogonial proliferation) and the *Zfy* transgenes under investigation. While the lack of the remaining Y chromosome gene content is known to have consequences during late spermiogenesis, previous work by us and others has shown that no other Y genes are necessary for progression through to and past the meiotic divisions, and therefore these genotypes are appropriate to investigate the effects of *Zfy* genes during the prophase of meiosis I ((Vernet et al., 2011; Vernet et al., 2014, Yamauchi et al., 2014).

At 17.5 dpp we observed widespread expression changes in *Zfy*-deficient testes that proved to be a combination of (a) MSCI leakage in early pachytene cells, and (b) loss of later-stage pachytene transcripts due to cell death during pachytene. We subsequently confirmed that the MSCI leakage is sustained beyond the first wave of juvenile spermatogenesis, and showed that transgenic restoration of *Zfy* genes corrected the MSCI leakage.

A parallel can be drawn with the situation in females, where unsynapsed chromatin is also silenced during oogenesis. In females, meiotic silencing is slower and leakier than in males : our results suggest that this may be due to the absence of *Zfy* genes in females. The genes involved in promoting meiotic silencing in females, and in promoting the delayed MSCI that occurs in X*^E^*O,*Sry* remain to be ascertained. Transgenic overexpression of *Zfx*, the X-linked homolog of *Zfy,* is able to compensate for *Zfy* deficiency in promoting the second meiotic division, and therefore these genes are functionally interchangeable to at least some extent (Vernet et al., 2014). *Zfx* is therefore an attractive candidate for a role in the less stringent MSCI observed in females and X*^E^*O,*Sry* males.

***Zfy*’s triple role at the MSCI checkpoint is suggestive of negative feedback regulation**

Previous work (Royo et al., 2010) established a role for *Zfy* genes as “executioners” at the MSCI checkpoint – to which we can now add a further role in the initial imposition of MSCI. A third role is implicit in their genomic location: since *Zfy* genes are Y-linked, *Zfy* transcription acts as a sensor for the success or failure of MSCI. Also implicit in the genomic location of *Zfy* is a negative feedback loop (which may be direct or indirect) in which *Zfy* genes repress their own expression at the transition into pachytene. The mechanism by which *Zfy* genes exert their effects remain to be elucidated, and it will be particularly important to establish the directness of the link between *Zfy* activity and the onset of MSCI, i.e. does it have a “preparatory” role prior to the onset of meiosis, or does it act in real-time to directly regulate its own expression?

If a real-time negative feedback loop can be demonstrated, this then suggests an elegant model in which in normal males, cells that are slow to achieve MSCI will continue to express *Zfy* genes, leading to one of two options. If the prolonged *Zfy* stimulus enables the lagging cells to complete MSCI, *Zfy* gene transcription ceases and prophase proceeds as normal. If MSCI remains incomplete even with prolonged *Zfy* gene activity, as in the various models with MSCI failure, then the cells undergo apoptosis. The same occurs if *Zfy* activity is artificially prolonged past the imposition of MSCI, as in the models with autosomal *Zfy* transgenes.

**The apoptotic response to MSCI leakage in X*^E^*O,*Sry* males is incomplete and age-dependent**

In this study, we find that during the first wave of spermatogenesis in X*^E^*O,*Sry* males, the apoptosis seen in association with MSCI leakage is incomplete and not restricted to stage IV, while in X*^E^*O,*Sry* males at 30-31 dpp there is no excess apoptosis of pachytene cells at any tubule stage despite ongoing MSCI leakage.

The first of these findings is unusual in that many models with MSCI failure show a strong stage IV block with virtually no spermatocytes surviving past stage IV. However, there are two other model systems that show MSCI leakage together with an incomplete stage IV block, these being *Ubr2* -/- males and *Trip13^mod/mod^* males . In both of these models, a variable proportion of spermatocytes survives past stage IV before succumbing to apoptosis, similar to the situation during the first wave of spermatogenesis in X*^E^*O,*Sry* males . The leakiness of the block in these males may be due to a low severity of MSCI disruption. In the H2AX null model, where MSCI is completely lost and there is complete apoptosis at stage IV, there is a twofold upregulation of X chromosome expression at both 16.5dpp and 18.5 dpp (Fernandez-Capetillo et al., 2003). In our data, we observe an average log_2_ ratio of 0.4 for X-linked genes at 17.5 dpp, i.e. only a 30% increase in X chromosome expression. Consistent with this hypothesis, the *Ubr2* knockout model (which also has an incomplete stage IV block) shows an average log_2_ ratio of 0.4 for X-linked genes at 17dpp . Expression profiling has not yet been performed on the *Trip13^mod/mod^* males at this age and so the level of MSCI leakage cannot be directly compared.

The findings in older X*^E^*O,*Sry* males– of ongoing MSCI leakage without a detectable increase in apoptosis during pachytene – are to our knowledge unprecedented. Logically there are two potential explanations for the difference between the first wave phenotype versus later ages: either there is less MSCI leakage in the spermatocytes of older animals, or the spermatocytes of older animals are better able to tolerate MSCI leakage without becoming apoptotic. It is not possible to distinguish these possibilities with current technology, since it would require tubule stage specific expression profiling of spermatocytes at each age.

**What can the models with MSCI “leakage” and an incomplete stage IV block tell us about MSCI mechanisms?**

Mechanistically, the MSCI leakage we observed in X*^E^*O,*Sry* spermatocytes was distinct from most other models with MSCI failure in that the cells nevertheless formed a morphologically normal sex body positive for γH2AX (**Figure 3 inset**). Intriguingly, this also applies to both *Trip13^mod/mod^* males and *Ubr2* -/- males, consistent with the low level of X upregulation in the latter and the incomplete stage IV apoptosis in both models. It is possible that the “leaky” genes in each case are contained within pockets of γH2AX-negative chromatin, however an alternative possibility is that the leakage reflects an alteration in silencing pathways operating downstream of, or independently to the standard BRCA1/ATR/γH2AX pathway of MSCI. Regardless of the precise mode of MSCI escape, these observations highlight that formation of a γH2AX-positive sex body must not be taken as proof of successful MSCI, and the only conclusive way to demonstrate sex chromosome silencing is by direct RNA FISH measurement of transcription.

**Evidence for multiple “executioner” genes at the stage IV checkpoint**

Four independent lines of evidence have led to the current view that there is an MSCI checkpoint acting during spermatogenesis. Firstly, many models with synapsis failure also show failure of MSCI, continued expression of *Zfy* genes during pachytene, and stage IV apoptosis . Secondly, in T(X;16)16H males (Searle’s translocation), those cells which fail to silence the X-derived portion of the X;16 translocation product undergo apoptosis during pachytene despite the fact that *Zfy* is correctly silenced in these cells . Thirdly, transgenic expression of either *Zfy1* or *Zfy2* during pachytene has been shown to trigger apoptosis of spermatocytes at tubule stage IV . Finally, transgenic expression of selected X-linked miRNA genes during pachytene leads to apoptosis at tubule stages IV and XII .

Collectively, these data suggest that both X-and Y-linked genes are independently capable of triggering apoptosis and acting as “executioner” genes for the MSCI checkpoint. Our data corroborate this and provide further evidence for the existence of multiple sex-linked executioner genes acting during pachytene, since the X*^E^*O,*Sry* males showed increased apoptosis of pachytene cells during the first wave of spermatogenesis in conjunction with MSCI leakage despite a complete absence of *Zfy* genes.

We note however that in T(X;16)16H males the stage at which aberrantly synapsed cells are eliminated is not known , and that overexpression of X-linked miRNAs triggered apoptosis at two different tubule stages . It is therefore not proven whether the apoptosis seen in response to wrongful X gene expression in these two models acts via the same mechanism as the *Zfy*-driven apoptosis occurring at tubule stage IV in other models of MSCI failure. Moreover, the extent and stage-specificity of the apoptosis in X*^E^*O,*Sry* males differs from that seen in most other models of MSCI failure. It may be that different executioner genes at the MSCI checkpoint trigger apoptosis via different downstream pathways and/or at different tubule stages, with *Zfy* simply being an early-acting executioner. Under this hypothesis, the timing of cell death during pachytene would depend not only on the degree of MSCI failure, but also on the specifics of which genes escape silencing in each cell. Like Tolstoy’s families, each dying spermatocyte in these males may be unhappy in its own way.

**Materials and Methods**

All animal procedures were in accordance with the United Kingdom Animal Scientific Procedures Act 1986 and were subject to local ethical review.

**Chromosome spreads**

Testes were frozen in liquid nitrogen and stored at -80 prior to thawing in RPMI medium. Surface spreads were prepared as described by Barlow et al and RNA FISH was performed as described previously .

**Fluorescence in situ hybridization (FISH)**

Surface spreads and fluorescence in situ hybridization (FISH) RNA-FISH for nascent nuclear transcripts from *Scml2*, *Zfx*, *GM773* and *Magea 2/5* was performed as previously described using spread testis cells from 6 week old X*^E^*O,*Sry*, X*^E,Z2^*O,*Sry* and XY MF1 male mice.

**Histological analysis and immunohistochemistry**

For standard histological analysis testes were fixed in Bouin’s fluid for 24 h, embedded in paraffin, sectioned at 5 μm on glass slides and then stained with Hematoxylin and Eosin (H & E). At least three males per genotype and age group were analyzed. For immunohistochemistry, testes were fixed overnight in 4% buffered paraformaldehyde at 4 degrees. Tissues were washed in 70% ethanol, dehydrated, and embedded in paraffin. 5μm sections on glass slides were dewaxed in xylene and hydrated in a graded series of alcohols. After washing in PBS, sections were blocked for an hour at room temperature in PBT (PBS, 0.1% Tween, 0.15% BSA) and incubated overnight at 37°C with primary antibody diluted in PBT. Slides were washed in PBS, incubated with appropriate fluorescently-conjugated secondary antibodies diluted in PBS for 1 h at 37°C, washed in PBS, and mounted in Vectashield containing 4’,6-diamidino-2-phenylindole (DAPI, Vector).

Further details of reagents used including BAC probe IDs and antibodies used for RNA FISH and immunostaining, and primers used for qPCR are given in **Supplementary Methods.**

**BrdU and TUNEL assays**

BrdU (Sigma), dissolved in phosphate-buffered saline, was injected intraperitoneally into males at 18-21 dpp, at 50 mg/kg of body weight. Males were killed 12 days after the injection, and testes were fixed in Bouin’s fluid for 24 hr, and then embedded in paraffin. BrdU incorporation was detected by immunofluorescence labeling as previously described . DAPI counterstaining, γH2AX immunostaining of spermatocytes, and PNA-lectin staining of the acrosomal cap (where present) were used to determine the stage of BrdU-positive seminiferous tubules. Phospho-histone H3 immuno-fluorescence and detection of apoptotic cells by TUNEL were performed as previously described .

**Quantitation of apoptosis**

At 15dpp apoptosis was quantified by hematoxylin and eosin (H&E) staining of histological sections. Since this age is prior to the MI arrest / apoptosis seen in X*^E,Z2^*O,*Sry* and the accumulation of arrested secondary spermatocytes in X*^E^*O,*Sry*, it therefore specifically measures cell death during meiotic prophase I. At 30-31 dpp, there are two confounding factors affecting the comparison of apoptosis between the two genotypes. Firstly, in stage XII tubules there is widespread apoptosis of cells at the spindle assembly checkpoint in X*^E,Z2^*O,*Sry* that is less pronounced in X*^E^*O,*Sry* . Secondly, in X*^E^*O,*Sry*, cells which fail to die at stage XII develop into diploid spermatids and subsequently undergo apoptosis throughout the following tubule stages, which will inflate the numbers of apoptotic cells per tubule in X*^E^*O,*Sry* . We therefore used three different methods to assay apoptosis in these males at d30-31.

1) Counting of apoptotic pachytene cells specifically in stage IV tubules in H&E sections. In these sections, tubules were staged by the hematoxylin staining pattern of the peripheral spermatogonia, and arrested diploid spermatids were distinguished by their nuclear morphology and excluded. This assay thus specifically measures cell death at the stage IV checkpoint.
2) Counting of TUNEL-positive cells in histological sections. In these preparations, detailed tubule staging was not possible and we therefore counted dying cells in all tubules from stages I – XI (defined as all tubules lacking meiotic metaphase figures). This analysis thus excludes the apoptotic metaphase I cells found in X*^E,Z2^*O,*Sry* at stage XII, but does not exclude the dying diploid spermatids seen in other stages in X*^E^*O,*Sry*. Accordingly, this comparison represents a very stringent check of increased apoptosis in X*^E^*O,*Sry*.
3) Counting the total number of meiosis I metaphase figures per stage XII tubule. TUNEL staining and staining for phospho-histone H3 was used to classify the metaphases as healthy or apoptotic, as previously described . This assay measures the total number of cells surviving through to meiosis I, and thus is an indirect measure of apoptosis occurring at all stages of meiotic prophase I.

**RNA extraction and expression analysis**

Total RNA was extracted from frozen testis tissue using Trizol (Invitrogen) according to the manufacturer’s protocol. Microarray analyses were performed as previously described using total testis RNA from males at 17.5 dpp. Briefly, single-color hybridization data (Illumina BeadChip, mouse whole-genome array, v2) was obtained for three X*^E^*O,*Sry* individuals and matched X*^E^*^,^*^Z2^*O,*Sry* controls, each individual being hybridized separately. Quantile normalization and differential expression testing was performed using BeadStudio (Illumina), and false discovery rate controlled using Benjamini and Hochberg FDR correction.

Quantitative RT-PCR experiments were performed as previously described . Briefly, two micrograms of total RNA were DNaseI-treated (Invitrogen), and reverse transcription of polyadenylated RNA was performed with Superscript Reverse Transcriptase II according to the manufacturer's protocols (Invitrogen). Samples from three 13.5, 15.5 and 17.5 day old mice for each genotype (X*^E^*O,*Sry* and X*^E,Z2^*O,*Sry*) were analyzed. All reactions were carried out in triplicate and beta actin was included on every plate as a loading control. The difference in PCR cycles with respect to beta actin (ΔCt) for a given experimental sample (X^E^O,*Sry*) was subtracted from the mean ΔCt of the reference samples (X*^E,Z2^*O,*Sry*) (ΔΔCt).

**Funding**

The work was funded by the Medical Research Council UK (PSB: U117532009, NV: MRC CDF) and EMBO (NV), and by the BBSRC (P.E., grant numbers BB/F007434/1 and BB/J00877X/1).

**Acknowledgments**

The authors thank Ms Aine Rattigan and Mr Obah A. Ojarikre for assistance with genotype analyses and mouse colony management respectively, Cambridge Genomic Services for microarray expression profiling, and Dr James Turner for helpful discussions of the results.

**Conflict of Interest Statement**

The authors declare that there are no conflicts of interest in regard to the work presented here.

**Author Contributions**

Conceptualization, P.S.B. and P.J.I.E.; Methodology, N.V., P.J.I.E and P.S.B..; Investigation, N.V., S.K.M., D.G.de R., and P.J.I.E.; Writing – Original Draft, P.J.I.E.; Writing – Review & Editing, N.V., D.G.de R., P.S.B. and P.J.I.E.; Funding Acquisition, P.S.B. and P.E.; Resources, N.V. and P.S.B.; Supervision, D.G.de R., P.S.B. and P.J.I.E.

**Data availability**

Normalised array data and the processing pipelines for figure generation are available in **Supplementary Table S1.** Raw array data is available through GEO, accession number XXXXX.

Tables

|  |  | **Proportion of tubules with at least 1 apoptotic cell** | **P value** | **Number of apoptotic cells per tubule with apoptosis** | **P value** | **Total number of cells per tubule** | **P value** |
| --- | --- | --- | --- | --- | --- | --- | --- |
| **15dpp, H&E staining, all tubule stages combined** | **X*^E^*O,*Sry*** | 77.0% +/- 5.3% | 0.0629 ^a^ | 8.92 +/- 0.61 | 0.0059 ^b^ | 32.15 +/- 2.20 | 0.773 |
|  | **X*^E,Z2^*O,*Sry*** | 56.9% +/- 5.0% |  | 3.63 +/- 0.26 |  | 33.00 +/- 1.63 |  |
| **30-31 dpp, H&E staining, stage IV tubules only** | **X*^E^*O,*Sry*** | 31.9% +/- 0.7% | 0.698 | 2.62 +/- 0.63 | 0.239 | n/d | n/d |
|  | **X*^E,Z2^*O,*Sry*** | 29.3% +/- 7.6% |  | 1.62 +/- 0.36 |  | n/d |  |
| **30-31dpp, TUNEL staining, tubule stages I - XI combined** | **X*^E^*O,*Sry*** | 39.7% +/- 4.5% | 0.121 | 2.71 +/- 0.1 | 0.204 | n/d | n/d |
|  | **X*^E,Z2^*O,*Sry*** | 28.9% +/- 3.1% |  | 2.48 +/- 0.12 |  | n/d |  |
| **30-31dpp, TUNEL staining, tubule stage XII, metaphase cells only** | **X*^E^*O,*Sry*** | 87.0% +/- 7.6% | 0.477 | 4.72 +/- 0.55 | 0.023 ^c^ | 10.41 +/- 1.72 | 0.677 ^d^ |
|  | **X*^E,Z2^*O,*Sry*** | 81.6% +/- 5.4% |  | 7.45 +/- 0.53 |  | 11.22 +/- 0.54 |  |

**Table 1:** Quantification of apoptosis in juvenile and older X*^E^*O,*Sry* and X*^E,Z2^*O,*Sry* males. An extended version of this table including raw tubule and counts is available as **Supplementary** **Table S3**.

^a^ Borderline significant increase in proportion of tubules with apoptosis in X*^E^*O,*Sry*, t test p value = 0.0629 after arcsine transformation of percentages
^b^ Significant increase in number of apoptotic cells per tubule in X*^E^*O,*Sry*, t test p value = 0.0059
^c^ Significant increase in number of apoptotic metaphase cells per tubule in X*^E,Z2^*O,*Sry* due to reinstatement of the SAC, t test p value = 0.023
^d^ No significant difference between genotypes in the total number of metaphase cells per tubule, t test p value = 0.677

**Figures**

**Figure 1 (see also Supplementary Table S1)**

**A:** Histogram showing the log_2_ expression ratio (+/- s.e.m) per chromosome for X*^E^*O,*Sry* versus X*^E,Z2^*O,*Sry*, averaged across all genes expressed in testis at 17.5 dpp. * In the X*^E^*O,*Sry* : X*^E,Z2^*O,*Sry* comparison, the X chromosome is significantly upregulated relative to the autosomes, p = 1.94 x 10^-72^.

**B:** Line chart of log_2_ expression ratio per chromosome for X*^E^*O,*Sry* and X*^E,Z2^*O,*Sry* relative to wild type XY, averaged across all genes expressed in testis at 17.5 dpp. Values are shown for each genotype as a whole, and for individual replicates within each genotype. Grey = autosomes, black = X chromosome. ** In the X*^E^*O,*Sry* : XY comparison, the X chromosome is significantly upregulated relative to the autosomes, p = 2.59 x 10^-76^. *** In the X*^E,Z2^*O,*Sry* : XY comparison, the X chromosome is significantly upregulated relative to the autosomes, p = 0.0013.

**C:** Scatter plot showing the log_2_ ratio of pachytene spermatocyte transcription to spermatogonial transcription (X axis) versus the log_2_ expression ratio of X*^E^*O,*Sry* and X*^E,Z2^*O,*Sry* at 17dpp (Y axis).

**D:** Quantitative RT-PCR data showing ΔΔct values for three X-linked genes (*Rhox3a*, *Atp7a* and *Zfx*) and three autosomal pachytene-specific genes (*Tdrd6*, *Piwil2* and *Mad2l2*) at 13, 15 and 17 dpp. Bars represent the expression levels in X*^E^*O,*Sry* relative to X*^E,Z2^*O,*Sry*, normalized to beta actin. Upregulation of *Rhox3a* is significant from 13dpp onwards and upregulation of *Atp7a* from 15 dpp onwards. Downregulation of *Tdrd6* and *Piwil2* is significant from 15dpp onwards, and downregulation of *Mad2l2* at 17dpp only.

**E:** RNA FISH staining data for spread pachytene cells at 16-18 dpp. Immunostaining for γH2AX was used to identify pachytene spermatocytes. Detection of an RNA FISH signal for *Scml2* indicates failure of sex chromosome silencing.

**Figure 2**

These panels show H&E staining of testis sections at 15dpp and 30-31 dpp, with age, genotype and scale as indicated.
**Left panels: low magnification overview**. Gross testicular organization is unperturbed.
**Middle panels: stage IV tubules**. At 15dpp both genotypes show a mix of healthy and apoptotic pachytene spermatocytes, with a higher proportion of apoptotic cells in X*^E^*O,*Sry* testes. At 30-31dpp there is very little apoptosis in either genotype, and there is an accumulation of diploid spermatids near the lumen in X*^E^*O,*Sry* testes that is not seen in X*^E,Z2^*O,*Sry* testes.

**Right hand panels: stage VII-VIII tubules (15dpp) or stage XII tubules (30-31dpp).**
At 15dpp in X*^E^*O,*Sry* testes some spermatocytes are able to survive stage IV and enter apoptosis at later stages. X*^E,Z2^*O,*Sry* testes show a larger proportion of surviving spermatocytes with few apoptotic cells visible. At 30-31dpp, spermatocytes undergoing meiosis I are abundant in both genotypes. In X*^E^*O,*Sry* testes the SAC is nonfunctional and the meiotic cells will survive despite X chromosome univalence to become diploid spermatids as seen in the X*^E^*O,*Sry* 30-31dpp middle panel. In X*^E,Z2^*O,*Sry* testes the SAC is functional and the majority of dividing spermatocytes are undergoing apoptosis at metaphase of meiosis I.

Arrows – healthy pachytene spermatocytes
Red arrowheads – apoptotic pachytene spermatocytes
Open arrowheads – diploid spermatids
Open arrows – intermediate spermatogonia used to identify stage IV tubules
Dotted arrows – preleptotene/leptotene spermatocytes used to identify stage VII-VIII tubules
Circles – metaphase I cells used to identify stage XII tubules: black = healthy, red = apoptotic

**Figure 3** (see also **Supplementary Table S2**)

Histogram showing the percentage of cells with a positive RNA FISH signal for *Scml2* in males at 29 dpp and older, i.e. after the first wave of spermatogenesis is complete. Each bar represents a measurement of ~100 cells from a single individual, with genotypes and ages as given on the X axis. Experiments focusing on early pachytene cells, mixed pachytene cells or late pachytene cells are presented in separate groups. Different genotypes are coloured as a visual aid.

The *Zfy* gene complements of each genotype are as follows:

Wild type XY – both *Zfy1*and *Zfy2* are present

X*^E^*O,*Sry* – no *Zfy* genes present (Vernet et al, 2011)

X*^E,Z2^*O,*Sry* – *Zfy2* only is present, in the form of an X-linked transgene (Vernet et al, 2011)

X*^E,Z1/U^*O,*Sry* – *Zfy1* only is present, in the form an X-linked integrant of a BAC containing both *Zfy1*and *Ube1y* (Royo et al, 2010)

X^Sxra^O – both *Zfy1* and *Zfy2* are present (Cattanach et al., 1971; Burgoyne et al., 1992)

**Figure 4** (see also **Supplementary Table S2**)

Solid bars: percentage of *Adam3­*-negative early pachytene spermatocytes that showed RNA FISH signals for a range of different X-linked genes in adult (≥ 6 week old) testis for each genotype. Shaded bars: percentage of *Adam3­*-positive late pachytene spermatocytes showing RNA FISH signals for each X-linked gene in each genotype. Inset: example RNA FISH image of a late stage X*^E^*O,*Sry* spermatid showing RNA FISH signals for *Scml2* (green) and *Adam3* (white), γH2AX immunostaining of the sex body (red) and DAPI counterstaining of nuclear DNA (blue).

**Figure 5**

Example images showing BrdU staining of testis tubules 12 days post-injection. Left panels: DAPI staining (blue) to show nuclei. Centre panels: staining with anti-γH2AX (yellow) and PNA-lectin (red) to allow tubule staging. Right panels: staining with anti-BrdU to identify cells actively synthesizing DNA at the time of injection. In each case the tubule shown has a peripheral layer of spermatocytes strongly positive for γH2AX, indicative of leptotene cells at stage IX-X. In all genotypes the next layer of cells is is BrdU-positive and contains a punctate γH2AX-positive sex body. In wild type XY only, many elongating spermatids positive for PNA-lectin staining can be seen near the lumen. Some PNA-lectin positive diploid round spermatids are visible in X*^E^*O,*Sry*. These are mostly removed by apoptosis in X*^E,Z2^*O,*Sry*, leading to a smaller tubule diameter for this genotype.

**REFERENCES**

An, J.Y., Kim, E.A., Jiang, Y., Zakrzewska, A., Kim, D.E., Lee, M.J., Mook-Jung, I., Zhang, Y., and Kwon, Y.T. (2010). UBR2 mediates transcriptional silencing during spermatogenesis via histone ubiquitination. Proc Natl Acad Sci U S A *107*, 1912-1917.

Baarends, W.M., Wassenaar, E., van der Laan, R., Hoogerbrugge, J., Sleddens-Linkels, E., Hoeijmakers, J.H., de Boer, P., and Grootegoed, J.A. (2005). Silencing of unpaired chromatin and histone H2A ubiquitination in mammalian meiosis. Mol Cell Biol *25*, 1041-1053.

Barchi, M., Mahadevaiah, S., Di Giacomo, M., Baudat, F., de Rooij, D.G., Burgoyne, P.S., Jasin, M., and Keeney, S. (2005). Surveillance of different recombination defects in mouse spermatocytes yields distinct responses despite elimination at an identical developmental stage. Mol Cell Biol *25*, 7203-7215.

Barlow, A.L., Benson, F.E., West, S.C., and Hulten, M.A. (1997). Distribution of the Rad51 recombinase in human and mouse spermatocytes. EMBO J *16*, 5207-5215.

Baudat, F., Manova, K., Yuen, J.P., Jasin, M., and Keeney, S. (2000). Chromosome synapsis defects and sexually dimorphic meiotic progression in mice lacking Spo11. Mol Cell *6*, 989-998.

Burgoyne, P.S., Mahadevaiah, S.K., Sutcliffe, M.J., and Palmer, S.J. (1992). Fertility in mice requires X-Y pairing and a Y-chromosomal "spermiogenesis" gene mapping to the long arm. Cell *71*, 391-398.

Burgoyne, P.S., Mahadevaiah, S.K., and Turner, J.M. (2009). The consequences of asynapsis for mammalian meiosis. Nat Rev Genet *10*, 207-216.

Cattanach, B.M., Pollard, C.E., and Hawker, S.G. (1971). Sex-reversed mice: XX and XO males. Cytogenetics *10*, 318-337.

Cloutier, J.M., Mahadevaiah, S.K., ElInati, E., Nussenzweig, A., Toth, A., and Turner, J.M. (2015a). Histone H2AFX Links Meiotic Chromosome Asynapsis to Prophase I Oocyte Loss in Mammals. PLoS Genet *11*, e1005462.

Cloutier, J.M., Mahadevaiah, S.K., ElInati, E., Toth, A., and Turner, J. (2015b). Mammalian meiotic silencing exhibits sexually dimorphic features. Chromosoma.

Cocquet, J., Ellis, P.J., Yamauchi, Y., Mahadevaiah, S.K., Affara, N.A., Ward, M.A., and Burgoyne, P.S. (2009). The multicopy gene Sly represses the sex chromosomes in the male mouse germline after meiosis. PLoS Biol *7*, e1000244.

Decarpentrie, F., Vernet, N., Mahadevaiah, S.K., Longepied, G., Streichemberger, E., Aknin-Seifer, I., Ojarikre, O.A., Burgoyne, P.S., Metzler-Guillemain, C., and Mitchell, M.J. (2012). Human and mouse ZFY genes produce a conserved testis-specific transcript encoding a zinc finger protein with a short acidic domain and modified transactivation potential. Hum Mol Genet *21*, 2631-2645.

Fernandez-Capetillo, O., Mahadevaiah, S.K., Celeste, A., Romanienko, P.J., Camerini-Otero, R.D., Bonner, W.M., Manova, K., Burgoyne, P., and Nussenzweig, A. (2003). H2AX is required for chromatin remodeling and inactivation of sex chromosomes in male mouse meiosis. Dev Cell *4*, 497-508.

Ghyselinck, N.B., Vernet, N., Dennefeld, C., Giese, N., Nau, H., Chambon, P., Viville, S., and Mark, M. (2006). Retinoids and spermatogenesis: lessons from mutant mice lacking the plasma retinol binding protein. Dev Dyn *235*, 1608-1622.

Gorbsky, G.J. (2015). The spindle checkpoint and chromosome segregation in meiosis. FEBS J *282*, 2471-2487.

Hamer, G., Roepers-Gajadien, H.L., van Duyn-Goedhart, A., Gademan, I.S., Kal, H.B., van Buul, P.P., and de Rooij, D.G. (2003). DNA double-strand breaks and gamma-H2AX signaling in the testis. Biol Reprod *68*, 628-634.

Homolka, D., Jansa, P., and Forejt, J. (2012). Genetically enhanced asynapsis of autosomal chromatin promotes transcriptional dysregulation and meiotic failure. Chromosoma *121*, 91-104.

Hunt, P.A., and Hassold, T.J. (2002). Sex matters in meiosis. Science *296*, 2181-2183.

Koopman, P., Ashworth, A., and Lovell-Badge, R. (1991). The ZFY gene family in humans and mice. Trends Genet *7*, 132-136.

Kurahashi, H., Kogo, H., Tsutsumi, M., Inagaki, H., and Ohye, T. (2012). Failure of homologous synapsis and sex-specific reproduction problems. Front Genet *3*, 112.

LeMaire-Adkins, R., Radke, K., and Hunt, P.A. (1997). Lack of checkpoint control at the metaphase/anaphase transition: a mechanism of meiotic nondisjunction in mammalian females. J Cell Biol *139*, 1611-1619.

Mahadevaiah, S.K., Bourc'his, D., de Rooij, D.G., Bestor, T.H., Turner, J.M., and Burgoyne, P.S. (2008). Extensive meiotic asynapsis in mice antagonises meiotic silencing of unsynapsed chromatin and consequently disrupts meiotic sex chromosome inactivation. J Cell Biol *182*, 263-276.

Mahadevaiah, S.K., Costa, Y., and Turner, J.M. (2009). Using RNA FISH to study gene expression during mammalian meiosis. Methods Mol Biol *558*, 433-444.

Mardon, G., Luoh, S.W., Simpson, E.M., Gill, G., Brown, L.G., and Page, D.C. (1990). Mouse Zfx protein is similar to Zfy-2: each contains an acidic activating domain and 13 zinc fingers. Mol Cell Biol *10*, 681-688.

Mazeyrat, S., Saut, N., Grigoriev, V., Mahadevaiah, S.K., Ojarikre, O.A., Rattigan, A., Bishop, C., Eicher, E.M., Mitchell, M.J., and Burgoyne, P.S. (2001). A Y-encoded subunit of the translation initiation factor Eif2 is essential for mouse spermatogenesis. Nat Genet *29*, 49-53.

Monesi, V. (1965). Differential rate of ribonucleic acid synthesis in the autosomes and sex chromosomes during male meiosis in the mouse. Chromosoma *17*, 11-21.

Nagaoka, S.I., Hassold, T.J., and Hunt, P.A. (2012). Human aneuploidy: mechanisms and new insights into an age-old problem. Nat Rev Genet *13*, 493-504.

Namekawa, S.H., Park, P.J., Zhang, L.F., Shima, J.E., McCarrey, J.R., Griswold, M.D., and Lee, J.T. (2006). Postmeiotic sex chromatin in the male germline of mice. Curr Biol *16*, 660-667.

Pacheco, S., Marcet-Ortega, M., Lange, J., Jasin, M., Keeney, S., and Roig, I. (2015). The ATM signaling cascade promotes recombination-dependent pachytene arrest in mouse spermatocytes. PLoS Genet *11*, e1005017.

Page, D.C., Mosher, R., Simpson, E.M., Fisher, E.M., Mardon, G., Pollack, J., McGillivray, B., de la Chapelle, A., and Brown, L.G. (1987). The sex-determining region of the human Y chromosome encodes a finger protein. Cell *51*, 1091-1104.

Roig, I., Dowdle, J.A., Toth, A., de Rooij, D.G., Jasin, M., and Keeney, S. (2010). Mouse TRIP13/PCH2 is required for recombination and normal higher-order chromosome structure during meiosis. PLoS Genet *6*.

Romanienko, P.J., and Camerini-Otero, R.D. (2000). The mouse Spo11 gene is required for meiotic chromosome synapsis. Mol Cell *6*, 975-987.

Royo, H., Polikiewicz, G., Mahadevaiah, S.K., Prosser, H., Mitchell, M., Bradley, A., de Rooij, D.G., Burgoyne, P.S., and Turner, J.M. (2010). Evidence that meiotic sex chromosome inactivation is essential for male fertility. Curr Biol *20*, 2117-2123.

Royo, H., Seitz, H., ElInati, E., Peters, A.H., Stadler, M.B., and Turner, J.M. (2015). Silencing of X-Linked MicroRNAs by Meiotic Sex Chromosome Inactivation. PLoS Genet *11*, e1005461.

Shin, Y.H., Choi, Y., Erdin, S.U., Yatsenko, S.A., Kloc, M., Yang, F., Wang, P.J., Meistrich, M.L., and Rajkovic, A. (2010). Hormad1 mutation disrupts synaptonemal complex formation, recombination, and chromosome segregation in mammalian meiosis. PLoS Genet *6*, e1001190.

Taketo, T., and Naumova, A.K. (2013). Oocyte heterogeneity with respect to the meiotic silencing of unsynapsed X chromosomes in the XY female mouse. Chromosoma *122*, 337-349.

Turner, J.M. (2015). Meiotic Silencing in Mammals. Annu Rev Genet *49*, 395-412.

Turner, J.M., Mahadevaiah, S.K., Ellis, P.J., Mitchell, M.J., and Burgoyne, P.S. (2006). Pachytene asynapsis drives meiotic sex chromosome inactivation and leads to substantial postmeiotic repression in spermatids. Dev Cell *10*, 521-529.

Vernet, N., Mahadevaiah, S.K., Ojarikre, O.A., Longepied, G., Prosser, H.M., Bradley, A., Mitchell, M.J., and Burgoyne, P.S. (2011). The Y-encoded gene zfy2 acts to remove cells with unpaired chromosomes at the first meiotic metaphase in male mice. Curr Biol *21*, 787-793.

Vernet, N., Mahadevaiah, S.K., Yamauchi, Y., Decarpentrie, F., Mitchell, M.J., Ward, M.A., and Burgoyne, P.S. (2014). Mouse Y-linked Zfy1 and Zfy2 are expressed during the male-specific interphase between meiosis I and meiosis II and promote the 2nd meiotic division. PLoS Genet *10*, e1004444.

Wojtasz, L., Cloutier, J.M., Baumann, M., Daniel, K., Varga, J., Fu, J., Anastassiadis, K., Stewart, A.F., Remenyi, A., Turner, J.M.*, et al.* (2012). Meiotic DNA double-strand breaks and chromosome asynapsis in mice are monitored by distinct HORMAD2-independent and -dependent mechanisms. Genes Dev *26*, 958-973.

Yamauchi, Y., Riel, J.M., Stoytcheva, Z. and Ward, M.A. (2014). Two Y genes can replace the entire Y chromosome for assisted reproduction in the mouse. Science *343*, 69-72.

Yoshida, K., Kondoh, G., Matsuda, Y., Habu, T., Nishimune, Y., and Morita, T. (1998). The mouse RecA-like gene Dmc1 is required for homologous chromosome synapsis during meiosis. Mol Cell *1*, 707-718.
